# Is Response Assessment of Breast Cancer Bone Metastases Better with Measurement of ^18^F-Fluoride Metabolic Flux Than with Measurement of ^18^F-Fluoride PET/CT SUV?

**DOI:** 10.2967/jnumed.118.208710

**Published:** 2019-03

**Authors:** Gurdip K. Azad, Musib Siddique, Benjamin Taylor, Adrian Green, Jim O’Doherty, Joanna Gariani, Glen M. Blake, Janine Mansi, Vicky Goh, Gary J.R. Cook

**Affiliations:** 1 Cancer Imaging Department, School of Biomedical Engineering and Imaging Sciences, King’s College London, St. Thomas’ Hospital, London, United Kingdom; 2 Department of Oncology, Guys and St. Thomas’ Hospital NHS Foundation Trust, London, United Kingdom; 3 King’s College London and Guy’s and St. Thomas’ PET Centre, St. Thomas’ Hospital, London, United Kingdom; 4 Department of Molecular Imaging, Sidra Medicine, Doha, Qatar; and; 5 Geneva University Hospitals, Geneva, Switzerland

**Keywords:** breast cancer, bone metastases, heterogeneity, ^18^F-fluoride PET/CT

## Abstract

Our purpose was to establish whether noninvasive measurement of changes in ^18^F-fluoride metabolic flux to bone mineral (K_i_) by PET/CT can provide incremental value in response assessment of bone metastases in breast cancer compared with SUV_max_ and SUV_mean_. **Methods:** Twelve breast cancer patients starting endocrine treatment for de novo or progressive bone metastases were included. Static ^18^F-fluoride PET/CT scans were acquired 60 min after injection, before and 8 wk after commencing treatment. Venous blood samples were taken at 55 and 85 min after injection to measure plasma ^18^F-fluoride activity concentrations, and K_i_ in individual bone metastases was calculated using a previously validated method. Percentage changes in K_i_, SUV_max_, and SUV_mean_ were calculated from the same index lesions (≤5 lesions) from each patient. Clinical response up to 24 wk, assessed in consensus by 2 experienced oncologists masked to PET imaging findings, was used as a reference standard. **Results:** Of the 4 patients with clinically progressive disease (PD), mean K_i_ significantly increased (>25%) in all, SUV_max_ in 3, and SUV_mean_ in 2. Of the 8 non-PD patients, K_i_ decreased or remained stable in 7, SUV_max_ in 5, and SUV_mean_ in 6. A significant mean percentage increase from baseline for K_i_, compared with SUV_max_ and SUV_mean_, occurred in the 4 patients with PD (89.7% vs. 41.8% and 43.5%, respectively; *P* < 0.001). **Conclusion:** After 8 wk of endocrine treatment for bone-predominant metastatic breast cancer, K_i_ more reliably differentiated PD from non-PD than did SUV_max_ and SUV_mean_, probably because measurement of SUV underestimates fluoride clearance by not considering changes in input function.

See an invited perspective on this article on page 320.

The bone-specific tracer ^18^F-fluoride is a marker of osteoblast activity in metastatic bone deposits. Both sclerotic and lytic metastatic bone lesions are highly ^18^F-fluoride–avid (1) and show increased blood flow and metabolic flux (plasma clearance) to the bone mineral compartment (K_i_), allowing quantification of the regional kinetics of abnormal bone metabolism on ^18^F-fluoride PET/CT (2). K_i_ is related to histomorphometric measures of bone turnover (3,4), and its measurement by PET has been proposed as a valuable and feasible method for measuring changes in regional bone turnover as a result of treatment in skeletal metastases from breast cancer (5) and has also been evaluated in assessing response in bone metastases from prostate cancer (6). By accounting for delivery and extraction of ^18^F-fluoride, K_i_ appeals as a more discriminatory parameter for assessing treatment response of bone metastases rather than static measures such as SUV (7,8). Although SUV is one of the commonest and simpler methods for quantifying ^18^F-fluoride PET studies, requiring only a short static scan and thus averting the need for invasive arterial blood sampling and lengthy dynamic scans, regional delivery and metabolic activity may be affected by changes in tracer kinetics at other sites in the body. Thus, K_i_ is a potentially more accurate and discriminatory parameter because both the delivery (arterial input function) and the local bone metabolism (time–activity curve) are measured over time to calculate kinetic indices of local bone metabolism (9,10).

Most quantitative ^18^F-fluoride PET studies have been performed using 60-min dynamic scans whereby the bone activity curve is combined with an arterial input function and K_i_ is calculated using the Hawkins 3-compartment model (11–15) or other simplified methods such as the Patlak method (16–18). However, only one dynamic scan can be acquired after a single injection of ^18^F-fluoride with a limited field of view on the PET scanner, and the invasive nature of arterial blood sampling and the requirement for trained personnel make this procedure unappealing for routine use.

Different approaches that are simpler to implement have been applied to avoid arterial cannulation (19–22) and allow multiple lesions to be measured (23). For example, a semi–population-based input function method has previously been proposed whereby K_i_ is calculated by initially fitting a terminal exponential to the measurements of venous plasma concentration and then adding a population-based residual curve (24). The main advantages of this methodology are to allow calculation of K_i_ from static ^18^F-fluoride PET scans in multiple lesions without the need for arterial sampling and to allow a more physiologic measure of changes in bone turnover in response to treatment (23).

Previous osteoporosis studies have reported differences in SUV and plasma clearance between cortical and trabecular bones suggesting different effects of treatment at different sites of the skeleton (15,25,26). One study reported a much higher mean percentage change in K_i_ than in SUV (24% vs. 3%) in the lumbar spine in osteoporotic women treated with teriparatide, suggesting potentially higher uptake of injected dose at other skeletal sites (15).

We hypothesized that measurement of K_i_ is feasible using a static method with venous blood measurement and that K_i_ is more discriminatory than SUV_max_ or SUV_mean_ in treatment response assessment. The aim of this study was to compare ^18^F-fluoride K_i_, derived from a static method, (27) with SUV_max_ and SUV_mean_ in assessing the response of breast cancer bone metastases to endocrine therapy and to determine the level of correlation between the 2 methods. We also aimed to evaluate the effect of endocrine therapy on K_i_ in nonmetastatic cortical and trabecular sites in the skeleton.

## MATERIALS AND METHODS

### Participants

Twelve female breast cancer patients (mean age, 50.4 y; range, 40–79 y) with de novo (5 patients, 20 lesions) or progressive bone metastases (7 patients, 52 lesions) starting endocrine treatment were included. Apart from 2 patients who had small-volume lung and liver metastases, all other patients had bone-only disease. The endocrine treatments were letrozole (*n* = 7), tamoxifen (*n* = 3), and everolimus/exemestane (*n* = 2). ^18^F-fluoride PET/CT scans were acquired before and 8 wk after starting treatment. Two experienced oncologists masked to the PET findings determined clinical response (based on standard imaging, including bone scans and CT, and on clinical assessment, including pain scores, alkaline phosphatase, and carcinoma antigen 15-3) up to 24 wk after the start of treatment or until progression, whichever came first. This assessment was used as a reference standard. We categorized patients as having either clinically progressive disease (PD) or nonprogressive disease (non-PD). We chose to include and assess patients with partial response and stable disease together as non-PD because clinical management rarely differs in these 2 groups.

The study was approved by a Research Ethics Committee and the Administration of Radioactive Substances Advisory Committee, and all patients gave written informed consent at the time of recruitment.

### Blood Sampling

Venous blood samples (5 mL each) were acquired at 55 and 85 min after injection of ^18^F-fluoride. Two 0.2-mL aliquots from each blood sample were weighed and then counted on a 10-sample well counter (2470 Wizard2; PerkinElmer) previously cross-calibrated with the PET scanner using a standard calibration technique subject to daily quality control. The calibration process used ^18^F-FDG mixed with water in a 6-L phantom to a known activity concentration and scanned on the PET scanner. Ten 0.2-mL samples were taken from the phantom and counted on the well counter for 3 min, allowing the calculation of a conversion factor between the scanner and well counter measured in counts per second per activity concentration (kBq/mL). Whole-blood samples were then centrifuged for 5 min (6,000 rpm), and two 0.2-mL samples of plasma from each were also weighed and then counted in the well counter. The resulting counts per minute were converted to activity concentrations (kBq/mL) using a calibration factor.

### ^18^F-Fluoride PET/CT Image Acquisition and Reconstruction

^18^F-fluoride (mean, 228 ± 15 MBq) was injected intravenously, and scanning commenced after an uptake time of 60 min. Images were acquired from skull base to upper thighs with an axial field of view of 15.7 cm and an 11-slice overlap between bed positions, using a Discovery 710 PET/CT scanner (GE Healthcare). A low-dose CT scan (140 kV, 10 mA, 0.5-s rotation time, and 40-mm collimation) was performed at the start of imaging to provide attenuation correction and an anatomic reference. The PET scan duration was set to 3 min per bed position.

PET image reconstruction included standard scanner-based corrections for radiotracer decay, scatter, randoms, and dead time. Emission sinograms were reconstructed with a time-of-flight ordered-subset expectation-maximization algorithm (2 iterations, 24 subsets), with a 256 × 256 matrix and a gaussian postreconstruction smoothing filter of 4 mm in full width at half maximum, available from the manufacturer on the scanner front-end.

### Image Analysis

On ^18^F-fluoride PET/CT, we defined PD as an increase by at least 25% in K_i_, SUV_max_, or SUV_mean_. Non-PD included patients with a partial response (>25% decrease in K_i_, SUV_max_, or SUV_mean_) or stable disease (<25% increase or decrease), as adapted from the criteria of the European Organization for Research and Treatment of Cancer, with our acknowledgment that these criteria were originally described for ^18^F-FDG (28).

Up to 5 of the hottest (SUV_max_ ≥ 10) (29) and largest (≥1 cm) lesions were selected for analysis in each subject. SUV measurements were normalized to body weight. The lesion regions of interest (ROIs) were contoured on the static ^18^F-fluoride PET/CT scans using in-house software. The ROIs were outlined semiautomatically using an initial 40% of the maximum tumor pixel threshold around each metastasis followed by manual correction based on an oncologist and radiologist working in consensus. Tumor volumes were measured from the PET ROIs both at baseline and again at 8 wk after starting treatment. The same ROIs were used to estimate K_i_, SUV_max_, and SUV_mean_ in each lesion. K_i_, SUV_max_, and SUV_mean_ were also measured in nonmetastatic trabecular (center of L1 lumbar vertebra) and cortical (upper femoral shaft, 1 cm below the lesser trochanter) bone similar to the metastatic ROIs. If L1 contained a metastasis, the nearest normal vertebra was used as a nonmetastatic trabecular ROI. No patients had metastatic disease in the subtrochanteric left femur.

### K_i_ Analysis

Values of K_i_ in metastases were calculated from the static scan. Venous blood samples and a modified Patlak method of calculation were used as previously described by Siddique et al. (27,30) by applying an input function obtained by adding a population residual curve to the exponential, obtained from the 2 venous blood samples taken 55 and 85 min after injection. The population arterial input function was acquired from 10 postmenopausal women as described previously (19) and in the supplemental data (31–34) (supplemental materials are available at http://jnm.snmjournals.org).

K_i_, SUV_max_, and SUV_mean_ at baseline and at 8 wk, as well as percentage change in K_i_, SUV_max_, and SUV_mean_ from baseline (in patients with PD and non-PD), were used for statistical analyses.

### Statistical Analysis

Changes in K_i_, SUV_max_, and SUV_mean_ were expressed as percentage change from baseline. Data that were normally distributed were expressed as a mean and SD and compared using the paired *t* test, and data that were not normally distributed were log-transformed first, allowing a normal distribution. Correlations between the changes in K_i_ against SUV_max_ and SUV_mean_ were evaluated using the Pearson correlation coefficient. For all statistical tests, a *P* value of 0.05 or less was considered statistically significant.

## RESULTS

By the clinical reference standard, there were 4 patients with clinical PD (20 lesions) and 8 with non-PD (32 lesions). The values of K_i_, SUV_max_, and SUV_mean_ at baseline (12 patients, 52 lesions) and at 8 wk (the same 52 lesions), and the percentage change in K_i_, SUV_max_, and SUV_mean_, are shown in Table 1 and the supplemental data.

**TABLE 1:** Comparison of Tumor Parameters at Baseline and at 8 Weeks

Baseline				8 wk				Mean % change		
Mean tumor volume	K_i_	SUV_max_	SUV_mean_	Mean tumor volume	K_i_	SUV_max_	SUV_mean_	K_i_	SUV_max_	SUV_mean_
6.8	0.067	35.1	18.8	6.6	0.08	38.3	20.8	35.1	16.0	17.2

Data are for 52 tumors. Units are cm^3^ for tumor volume, mL min^−1^ mL^−1^ for K_i_, and g/mL for SUV.

Correlations were present between K_i_ and SUV_max_ and between K_i_ and SUV_mean_ at baseline (*r* = 0.632 [*P* < 0.001] and *r* = 0.784 [*P* < 0.001], respectively) and at 8 wk (*r* = 0.830 [*P* < 0.001] and *r* = 0.901 [*P* < 0.001], respectively) (Table 2).

**TABLE 2:** Correlation Between K_i_ and SUV at Baseline and at 8 Weeks

	Correlation coefficient				
			% change		
Comparison	Baseline	8 wk	All patients	PD patients	Non-PD patients
K_i_ vs. SUV_max_	0.632 (*P* < 0.001)	0.830 (*P* < 0.001)	0.852 (*P* < 0.001)	0.811 (*P* < 0.001)	0.863 (*P* < 0.001)
K_i_ vs. SUV_mean_	0.784 (*P* < 0.001)	0.901 (*P* < 0.001)	0.901 (*P* < 0.001)	0.904 (*P* < 0.001)	0.933 (*P* < 0.001)

Data are for 52 tumors in 12 patients. Units are mL min^−1^ mL^−1^ for K_i_ and g/mL for SUV.

**FIGURE 1.**
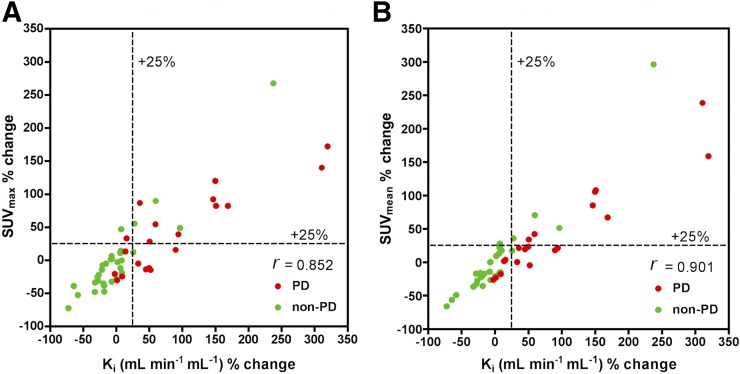
Lesion analysis: Scatterplot for percentage changes in K_i_ against percentage changes in SUV_max_ and SUV_mean_ for PD and non-PD 8 wk after start of treatment.

**FIGURE 2.**
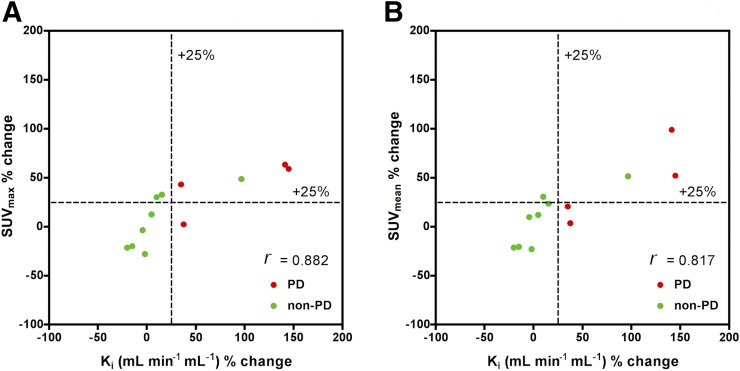
Patient basis: Scatterplot for percentage changes in K_i_ against percentage changes in SUV_max_ and SUV_mean_ for PD and non-PD 8 wk after start of treatment.

In all patients on a per-lesion basis, a statistically significant correlation was observed at 8 wk between percentage change in K_i_ and percentage change in SUV_max_ (*r* = 0.852, *P* < 0.001) and between percentage change in K_i_ and percentage change in SUV_mean_ (*r* = 0.901, *P* < 0.001) (Fig. 1; Table 2). In PD patients, a statistically significant correlation was observed at 8 wk between percentage change in K_i_ and percentage change in SUV_max_ (*r* = 0.811, *P* < 0.001) and between percentage change in K_i_ and percentage change in SUV_mean_ (0.904, *P* < 0.001). In non-PD patients, a statistically significant correlation was observed at 8 wk between percentage change in K_i_ and percentage change in SUV_max_ (*r* = 0.863, *P* < 0.001) and between percentage change in K_i_ and percentage change in SUV_mean_ (*r* = 0.933, *P* < 0.001) (Table 2). In all patients on a per-patient basis, a correlation was noted between mean percentage change in K_i_ and mean percentage change in SUV_max_ (*r* = 0.88, *P* < 0.001) and between mean percentage change in K_i_ and mean percentage change in SUV_mean_ (*r* = 0.81, *P* = 0.001) (Fig. 2).

Measurements of K_i_ after 8 wk of endocrine therapy showed a significant change in metastases from baseline, with an overall mean increase of 35.1% (SD, 58.4%) for K_i_, compared with 16.0% (SD, 32.4%) for SUV_max_ (*P* = 0.005) and 17.2% (SD, 35.7%) for SUV_mean_ (*P* = 0.001) (Table 1). In patients with PD, the mean percentage increase was 89.7% (SD, 61.7%) for K_i_, compared with 41.8% (SD, 27.8%) for SUV_max_ (*P* = 0.001) and 43.5% (SD, 41.9%) for SUV_mean_ (*P* < 0.001). In non-PD patients, the mean percentage increase was 11.0% (SD, 36.7%) for K_i_, compared with 6.2% (SD, 28.9%) for SUV_max_ (*P* = 0.60) and 7.4% (SD, 27.5%) for SUV_mean_ (*P* = 0.70). The mean percentage increase in K_i_ was statistically significantly higher in patients with PD than in patients with non-PD (K_i_ = 89.7% vs. 10.7%, *P* < 0.01), but the same was not true for SUV_max_ (41.8% vs. 6.2%, *P* = 0.067) or SUV_mean_ (43.5% vs. 7.4%, *P* = 0.153)) (Table 3).

**TABLE 3:** Comparison of Tumor Parameters at Baseline and at 8 Weeks in Individual PD and non-PD Patients

		Baseline			8 wk			% change		
Patient type	Patient no.	K_i_	SUV_max_	SUV_mean_	K_i_	SUV_max_	SUV_mean_	K_i_	SUV_max_	SUV_mean_
PD	1	0.033	25.7	12.0	0.075	37.1	16.9	145.0	58.8	51.8
	2	0.071	32.6	20.2	0.091	32.6	19.6	38.6	2.1	3.3
	3	0.047	49.6	21.9	0.121	85.9	46.8	140.2	63.2	98.4
	4	0.083	30.7	19.4	0.110	44.0	23.1	34.9	43.0	20.4
Non-PD	1	0.067	25.8	11.7	0.063	25.74	12.72	−4.7	−4.1	9.4
	2	0.145	60.9	34.4	0.121	46.96	26.64	−14.5	−20.0	−20.7
	3	0.078	37.6	21.7	0.076	26.70	16.72	−1.3	−28.0	−23.1
	4	0.045	38.8	19.8	0.036	30.38	15.47	−19.8	−21.7	−21.8
	5	0.051	26.1	14.4	0.051	27.35	15.40	5.4	12.3	11.4
	6	0.040	12.4	25.3	0.079	37.59	18.77	97.5	48.2	50.8
	7	0.055	25.0	14.8	0.063	34.73	18.31	15.6	32.5	23.0
	8	0.059	27.6	13.4	0.051	23.68	14.02	10.1	30.1	30.5
P*								*P* = <0.01	*P* = 0.067	*P* = 0.153

* PD vs. non-PD.

PD data are for 20 tumors; non-PD data are for 32 tumors. Units are mL min^−1^ mL^−1^ for K_i_ and g/mL for SUV.

On a per-lesion basis, K_i_ increased by more than 25% in 15 of 20 lesions (75%) (*P* = 0.13), SUV_max_ in 11 of 20 lesions (55%) (*P* = 0.13), and SUV_mean_ in 8 of 20 lesions (40%) (*P* = 0.02) in patients with clinical PD. K_i_, SUV_max_, and SUV_mean_ decreased or remained stable in 27 of 32 lesions (84%) in patients with clinical non-PD. K_i_, SUV_max_, and SUV_mean_ were falsely positive (increased >25%) in 5 of the 32 lesions in patients with clinical non-PD.

On a per-patient basis, of the 4 patients with clinical PD, mean percentage change in K_i_ correctly identified all 4, mean percentage change in SUV_max_ identified 3 of the 4, and mean percentage change in SUV_mean_ identified 2 of the 4. Of the 8 patients with clinical non-PD, mean percentage change in K_i_ accurately identified 7 of the 8, percentage change in SUV_max_ identified 5 of the 8, and percentage change in SUV_mean_ identified 6 of the 8. K_i_ was falsely positive in 1 of the 8 patients, SUV_max_ in 3 of the 8, and SUV_mean_ in 2 of the 8.

The mean percentage change in K_i_ was 7.9 times higher in patients with high disease burden (*n* = 7) (>5 bone metastases) than in those with low disease burden (*n* = 5) (48.8% vs, 6.2%, *P* = 0.017). There was no significant difference in mean percentage change in SUV_max_ (18.6% vs. 10.9%, *P* = 0.22) or mean percentage change in SUV_mean_ (20.3% vs. 11.7%, *P* = 0.12) between the 2 groups.

In normal cortical and trabecular nonmetastatic bone, the percentage change in K_i_ was 21.7% versus −1.9%, respectively; the percentage change in SUV_max_ was 4.8% versus −12.6%, respectively; and the percentage change in SUV_mean_ was −0.7% versus −17.1%, respectively. There was a statistically significant difference in K_i_ between baseline and 8 wk for cortical bone (*P* = 0.018) and a statistically significant difference in SUV_max_ between baseline and 8 wk for trabecular bone (*P* = 0.050), but other differences were not significant.

## DISCUSSION

To our knowledge, this was the first study reporting an advantage in measuring changes in metabolic flux (plasma clearance) of ^18^F-fluoride to bone mineral (K_i_) as an early, 8-wk, treatment response marker for breast cancer bone metastases compared with the static semiquantitative measures SUV_max_ and SUV_mean_. The methodology allows estimation of the arterial input function by correcting a population input function from venous plasma measurements. Together with measurements from a static PET acquisition at 60 min after injection, K_i_ can be estimated in any lesion within the static field of view (27).

We observed expected correlations between K_i_ and SUV parameters at baseline and at 8 wk and between percentage change in K_i_ and percentage change in SUV parameters in patients with PD or non-PD.

Compared with a clinical reference standard using data up to 24 wk, percentage change in K_i_ at 8 wk correctly predicted PD in more patients and more lesions than either SUV_max_ or SUV_mean_ (in 4, 3, and 2 of 4 patients, respectively, and in 15, 11, and 8 of 20 lesions, respectively) in patients with bone-predominant breast cancer undergoing endocrine treatment. K_i_ correctly predicted non-PD in more patients than SUV_max_ or SUV_mean_ (in 7, 5, and 6 of 8 patients, respectively) but no difference was observed on a per-lesion basis. The mean percentage increase in K_i_ was statistically significantly higher in patients with PD than in patients with non-PD.

The metabolic flux of ^18^F-fluoride provides an assessment of local bone mineralization taking into account the availability of tracer (i.e., input function), whereas measurements of SUV ignore possible changes in the input function. When plasma ^18^F-fluoride concentration is reduced, either because of a high global avidity of metastatic lesions or an increase in the metabolic activity of the remaining normal skeleton, then SUV parameters may underestimate mineralization in individual lesions. This possibility is supported by our observations. First, patients with a higher disease burden showed significantly greater changes in K_i_ than patients with a low disease burden. Second, we observed an increase in metabolic activity in the nonmetastatic skeleton at both trabecular and cortical sites, presumably as an effect of endocrine treatment. These changes were lower than those seen in metastases—thus maintaining contrast between metastatic and normal skeleton—but were greater when measured by K_i_ than by SUV parameters in cortical bone.

The relatively small number of patients in this study limits statistical comparisons, but this limitation was partly mitigated by a larger number of lesions (*n* = 52). Although measurement of K_i_ shows advantages over SUV_max_, false-positives caused by the flare phenomenon remain a factor that must be considered, as previously described (35–37). Our observation of more than a 25% increase in K_i_ in 5 of 32 lesions (in non-PD patients) might be accounted for by this phenomenon. Nevertheless, the ability to predict PD or non-PD after 8 wk of endocrine treatment remained good in this cohort, especially using K_i_, with all 4 of the PD patients and 7 of the 8 non-PD patients being correctly predicted, and K_i_ was a significantly better discriminator of PD from non-PD. Some of the smaller metastases might have been susceptible to partial-volume error, introducing potential bias, but we did not attempt to correct for this. Percentage change, rather than absolute values, of parameters was of primary interest, and partial-volume errors would have been similar for each parameter given that the same ROIs were used for calculations. Because the modified Patlak method corrects for ^18^F-fluoride efflux from bone, errors resulting from no direct measurement of backflow from bone mineral (*k*_4_) would be minimal (23). The population input function used in our method was derived from postmenopausal women, and although these did not have metastatic breast cancer, they did not have any other known skeletal disease and had a mean age similar to our patient cohort (54.8 and 50.4 y, respectively). In addition, it has previously been shown that precision errors in ^18^F-fluoride PET skeletal static and kinetic parameters are relatively small (coefficient of variation, 12%–14%) (38) and generally less than the changes we observed in this series.

Prediction of PD is most important to the oncologist by allowing an earlier transition to second- or third-line therapy while minimizing potential toxicity from ineffective treatment. Patients with either stable disease or a partial or complete response would normally have treatment continued; it is therefore clinically relevant to include both stable and responder groups together in this analysis.

A gold standard for predicting treatment response in bone metastases is lacking in clinical practice. However, our clinical reference standard was made as robust as possible by using standard clinical data (imaging, biochemistry, tumor markers, and clinical features) in consensus by 2 oncologists masked to the PET results and with the time advantage of allowing assessment up to 24 wk.

## CONCLUSION

This study has shown that measurement of ^18^F-fluoride metabolic flux (K_i_) in breast cancer bone metastases, using static ^18^F-fluoride PET/CT with venous blood sample counts, is feasible and may be more reliable in differentiating PD from non-PD than semiquantitative SUV measures. In particular, the observed accurate identification of PD is important and of clinical utility. These preliminary results deserve further prospective validation in larger patient groups under different therapy regimes.

## DISCLOSURE

Financial support was received from the King’s College London/University College London Comprehensive Cancer Imaging Centres funded by Cancer Research U.K. and the Engineering and Physical Sciences Research Council in association with the Medical Research Council and the Department of Health (C1519/A16463), Breast Cancer Now (2012NovPR013), the Wellcome Trust EPSRC Centre for Medical Engineering at King’s College London (WT203148/Z/16/Z), the Royal College of Radiologists, Alliance Medical Ltd., and the National Institute of Health Research Clinical Research Network (NIHR CRN). No other potential conflict of interest relevant to this article was reported.
